# Gender-Related Differences in Prodromal Multiple Sclerosis Characteristics: A 7-Year Observation Study

**DOI:** 10.3390/jcm10173821

**Published:** 2021-08-26

**Authors:** Jakub Perwieniec, Krzysztof Podwójcic, Michał Maluchnik, Mateusz Szeląg, Dorota Walkiewicz, Michał Zakrzewski, Amelia Droździkowska, Bogumił Kamiński, Adriana Zasybska, Marcin Wnuk, Agnieszka Słowik, Konrad Rejdak

**Affiliations:** 1 Department of Analysis and Strategy, Ministry of Health of the Republic of Poland, 00-952 Warsaw, Poland; jakubperwieniec@gmail.com (J.P.); m.szelag@mz.gov.pl (M.S.); d.walkiewicz@mz.gov.pl (D.W.); m.zakrzewski@mz.gov.pl (M.Z.); a.drozdzikowska@mz.gov.pl (A.D.); 2 Institute of Labour and Social Studies, 01-022 Warsaw, Poland; k.podwojcic@gmail.com; 3 Department of Adult Neurology, Medical University of Gdańsk, 80-211 Gdańsk, Poland; m.maluchnik@mz.gov.pl; 4 SGH Warsaw School of Economics, 02-554 Warsaw, Poland; bkamins@sgh.waw.pl; 5 Department of Neurology, Medical University of Lublin, 20-090 Lublin, Poland; adriana.zasybska@umlub.pl; 6 Department of Neurology, Jagiellonian University Medical College, 30-688 Krakow, Poland; marcin.wnuk@uj.edu.pl (M.W.); agnieszka.slowik@uj.edu.pl (A.S.); 7 Department of Neurology, University Hospital in Krakow, 30-688 Krakow, Poland

**Keywords:** multiple sclerosis, risk factors, pre-existing diseases, pre-existing conditions, Poland, administrative data

## Abstract

Increasing evidence supports the observation that multiple sclerosis (MS) has a preclinical period, with various prodromal signs and symptoms more frequently represented in patients with confirmed MS many years later. Considering the apparent gender differences in the incidence and clinical course of MS, it remains unclear whether it could be reflected in prodromal symptom features. This study aimed to compare a broad spectrum of prodromal signs and symptoms between males and females in the 7-year period before the definite diagnosis of MS. Data came from the central register of the national payer of services, financed under the public healthcare system in Poland. They covered a 7-year period of patient health record claims, from 2009 to 2016. The following groups of symptoms were significant with women: musculoskeletal (*p* < 0.001), ophthalmic (*p* < 0.001), laryngological (*p* < 0.001), digestive system (*p* < 0.001), urinary tract (*p* < 0.001), mental (*p* < 0.001), cardiovascular (*p* < 0.001), complaints and headaches (*p* < 0.001). There was also a weak correlation with head injuries (*p* = 0.03) while dermatological and reproductive system complaints did not appear to be significant (*p* < 0.05). For males, the following groups of symptoms were significant: musculoskeletal (*p* < 0.001), ophthalmic (*p* < 0.001), laryngological (*p* = 0.007), cardiovascular system symptoms (*p* < 0.001), and headaches (*p* < 0.001). Interestingly, reproductive system problems were overrepresented in the male population (*p* = 0.008). There was no significant correlation with MS risk for dermatological, digestive, urinary, and mental complaints. Similarly, head injuries were not significant. Our results shed more light on well-known differences in the epidemiological and clinical characteristics between sexes in multiple sclerosis, and show differences in prodromal complaints before MS onset.

## 1. Introduction

Increasing evidence supports the observation that MS has a preclinical period, with various prodromal signs and symptoms more frequently represented in patients with confirmed MS many years later [1,2,3,4]. Based on data from linked health administrative and clinical databases from four Canadian provinces, annual healthcare use increased steadily, anywhere between 5 years and 1 year before the first demyelinating disease claim in people with multiple sclerosis compared with controls for hospital admissions, physician claims, and prescriptions, assessed as drug classes [5]. MS patients in the UK population had a significantly higher risk of presenting with gastric, intestinal, urinary, and anorectal disturbances, anxiety, depression, insomnia, fatigue, headache, and various types of pain among patients, up to 10 years prior to the index date [1]. Another population-based matched cohort study using linked administrative and clinical databases was performed on the Canadian population [6]. It confirmed that fatigue, sleep disorders, anaemia, and pain were more frequent among MS cases relative to controls. The association between MS and anaemia was greater in men, while that between MS and pain increased with age. The same Canadian group compared relapsing-remitting and primary-progressive patients in that respect, concluding that both may experience a prodrome, although clinical aspects may differ [2].

There is a great need to extend such studies to characterize MS prodrome in detail, as it can have implications in better understanding the true nature of the disease, including risk factors and pathogenesis. Considering the apparent gender differences in the MS incidence and clinical course [7], it remains unclear whether it can be reflected in the prodromal symptom features.

This study aims to compare a broad spectrum of signs and symptoms resulting from different system and organ dysfunctions between males and females in the 7-year period before the definite diagnosis of MS.

## 2. Materials and Methods

Data came from the central nationwide register of the payer of services, financed under the public healthcare system—the National Health Fund (NFZ) electronic database. They covered a 7-year period of patient health record claims, from 2009 to 2016. Based on the analysis of the history of provided services, two groups of patients were selected: (i) those with multiple sclerosis (MS) diagnosed in 2016 who were aged 1–86 years (3056 people) and (ii) a random (control) sample of 300,000 patients, aged 1–86 from the population of all 2016 patients in the database (Table 1).

MS cases were confirmed if a patient made at least two visits to outpatient specialist care, inpatient care, or outpatient rehabilitation services with a G35 diagnosis code within 3 years from the first G35 diagnosis, and at least one visit with a G35 diagnosis at a neurological ward (including paediatric clinics), or who participated in a disease-modifying-therapy (DMT) programme designed for MS patients. In addition, the end of an MS prodromal period was the date of the patient’s first demyelinating event (medical appointment with a G35 code). Ultimately, the analysed MS sample consisted of 3056 patients aged 1–86 years, including 2128 females and 928 males.

The matched control group included 300,000 individuals aged 1–86, of whom 208,889 were female and 91,111 male. Additionally, a random stratified draw from the whole non-MS population was carried out—the sample’s age and sex structures were identical to the age and sex of the MS population (matched by age and sex). 

For the pooled sample, medical history records were acquired. Based on initial exploratory data analysis, patients’ records were aggregated into 11 homogenous groups according to a key showed in Table 2. Patients’ episodes explored (MS or non-MS) before the first G35 diagnosis lasted equally 7 years. 

Individuals were assigned a particular condition if at least one ICD-10 code within a given disease group was reported during the prodromal episode (2009–2016). Based on such classification, univariate analysis was carried out. For every examined condition, incidences among MS cases and control groups were evaluated and compared. In addition, differences of incidences in a whole population as well as within gender-specific groups were investigated. Thus, relative risk measure-risk ratio (RR) estimates were calculated with 95% confidence intervals (using the Wald normal approximation). Moreover, based on 2 × 2 contingency table values for every analysed condition, the test of independence two-sided *p*-values was performed using Fisher’s exact test. Furthermore, for every explored group of symptoms, differences of risk in intervals of 365 days (determined by the patient-specific index date) were found. All database querying, data processing, and computations, as well as visualisations, were carried out using GNU R programming language within the RStudio environment.

## 3. Results

### 3.1. Total MS Population Prodromal Symptom Analysis

Musculoskeletal diseases were the most common prodromal symptom group among patients with MS, reported by nearly 60% of all patients (see Table 3). These symptoms occurred in a control group in 48.2% of patients (risk ratio (RR): 1.548; 95% RR confidence intervals: (1.44, 1.663); Fisher’s exact test *p*-value: <0.001). The second most frequently reported issues were ophthalmic diseases, which occurred in 49.9% of MS patients and 35.1% of controls (RR: 1.830 (1.706, 1.964); *p* < 0.001). Symptoms of dermatological diseases were the third most often observed conditions (40.6% of MS patients and 39.1% of controls). However, the difference between the MS group and the control individuals was not statistically significant (RR: 1.065 (0.991, 1.144); *p* < 0.09). The biggest difference between MS patients and general population controls was observed for the headache-related group of symptoms—30% of MS patients and 16.8% of controls (RR: 2.176 (2.016, 2.348); *p* < 0.001). Moreover, the highest increase of risk of MS incidence was noted for individuals diagnosed with ophthalmic diseases, musculoskeletal diseases, cardiovascular diseases (RR: 1.469 (1.344, 1.605); *p* < 0.001), and laryngological diseases (RR: 1.411 (1.301, 1.531); *p* < 0.001). Moreover, patients reporting urinary (RR: 1.255, (1.155, 1.365); *p* <0.001) and digestive (RR: 1.109 (1.031, 1.194); *p* = 0.006) symptoms were more likely to subsequently develop multiple sclerosis. Presuming a significance level of 0.05, estimates for reproductive system diseases (RR: 1.049 (0.976, 1.128); *p* = 0.196) and head injuries (RR: 1.252 (0.948, 1.653); *p* = 0.128) suggested a lack of association between their occurrence during the prodromal period and MS incidence. 

### 3.2. Gender-Related Prodromal Symptom Analysis

In the subgroup analysis, we compared the occurrence of prodromal symptoms separately for females and males compared to the matched controls from the general population. With regard to the female subgroup, we observed that musculoskeletal diseases were the most common prodromal group of symptoms, present in 62.4% of surveyed MS patients versus 49.8% of controls (Table 3). Ophthalmic diseases were diagnosed in 54.1% of MS female patients and only in 38.6% of controls. The third most frequent group of problems were reproductive system issues, observed among nearly half of investigated subjects (49.9% of MS cases and 49.5% of controls). However, reported dermatological diseases and reproductive system issues did not increase the risk of MS incidence; risk ratio values were not significantly different than 1 (risk ratio of 1.067 (0.98, 1.162); *p* = 0.134 and 1.016 (0.933, 1.105); *p* = 0.727, respectively). Major MS risk differences between female subpopulations were found for headaches (RR: 2.231 (2.043, 2.436); *p* < 0.001), ophthalmic diseases (RR: 1.862, (1.711, 2.027); *p* < 0.001) and musculoskeletal diseases (RR: 1.666 (1.527, 1.818); *p* < 0.001). The incidence of the rest of the examined conditions significantly raised the chances of developing MS. Nevertheless, statistically significant differences were observed for laryngological diseases (RR: 1.477 (1.343, 1.625); *p* < 0.001), digestive system diseases (RR: 1.172 (1.075, 1.278); *p* < 0.001), urinary tract diseases (RR: 1.274 (1.16, 1.399); *p* < 0.001), mental illnesses (RR: 1.339 (1.216, 1.473); *p* < 0.001), cardiovascular diseases (RR: 1.508 (1.356, 1.676); *p* < 0.001), and head injuries (RR: 1.508 (1.055, 2.157), *p* = 0.031). Dermatological and reproductive system problems were not significant. 

In the male subpopulation, the most common symptoms were musculoskeletal diseases (51.7% of MS cases and 44.7% of controls) and ophthalmic diseases (40.2% of MS cases and 27% of controls). Dermatological diseases (32.4% of MS cases and 31.2% of controls) were the third most frequent conditions, but the risk difference of MS incidence for those patients turned out to be statistically insignificant (RR: 1.060 (0.925, 1.215); *p* = 0.413). Generally, lower incidence figures in the male subgroup were observed.

The greatest differences between the male MS patient group and the male control group were observed for headaches (RR: 1.93 (1.815, 2.505); *p* < 0.001), ophthalmic symptoms (RR: 1.808 (1.587, 2.060); *p* < 0.001), and cardiovascular complaints (RR: 1.382 (1.173, 1.628); *p* < 0.001). The symptom groups, which did not significantly differ from the general population, included dermatological diseases (RR: 1.060 (0.925, 1.215); *p* = 0.413), digestive system problems (RR: 0.963 (0.836, 1.109); *p* = 0.613), urinary tract symptoms (RR: 1.223 (0.995, 1.503); *p* = 0.057), mental illnesses (RR: 1.030 (0.841, 1.262); *p* = 0.751), as well as head injuries (RR:0.996 (0.641, 1.547); *p* = 1.0) at α = 0.05 significance level.

**Figure 1:**
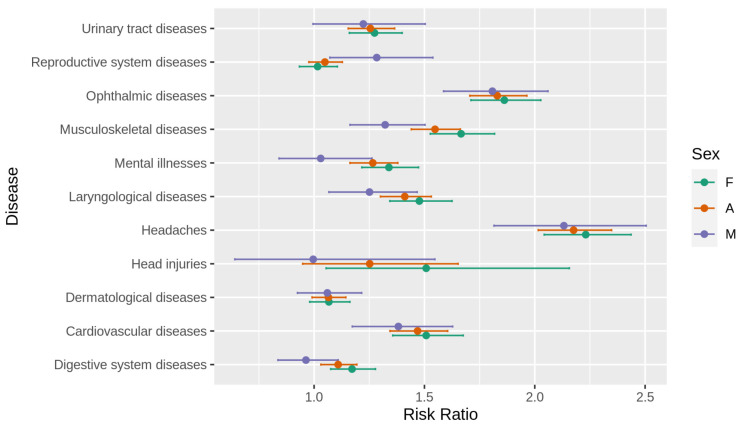
Risk ratio with corresponding 95% confidence intervals for analysed symptoms. F—female, M—male, A—all.

Full results of the aforementioned univariate analysis (risk ratio estimation) of the difference between the MS population and the random population for both gender-specific subgroups, as well as the pooled population, are visualised in Figure 1. Detailed values of analysis outcome are presented in Table S1 (attached in Supplementary Materials). All underlying incidence information is demonstrated in Table 3.

Investigating conditions in which incidence may be considered MS prodromal symptoms, we also focused on the time when the symptoms occurred (with respect to the index date—the first demyelinating event). For most condition groups that were validated as statistically significant, we observed different dynamics of the manifestation of symptoms—a considerable increase in the risk ratio occurring during the last years before the first G35 code diagnosis, with a peak value during the last year before the index date. However, for those with certain conditions, such as musculoskeletal diseases or headaches reported by women, the risk of developing MS was statistically significantly higher even seven years before diagnosis. Temporal risk ratio estimates of all examined conditions for analysed subgroups are presented in Figures S1–S11 (see Supplementary Materials). Relatively less accurate estimates (visualised with broader confidence intervals) for male patients resulted from a smaller sample size.

## 4. Discussion

This study focused on gender-related differences in prodromal signs and symptoms of MS in the period of 7 years before a clinically confirmed diagnosis.

We identified several features acting as possible prodrome or risk factors more frequently represented before the full-blown MS.

Firstly, we confirmed previous findings identifying a list of different symptoms more frequently encountered in the preclinical period before MS onset compared to the general population [1,2,8]. They included headaches, ophthalmic, musculoskeletal, cardiovascular, digestive, urinary, laryngological, and mental complaints. In particular, MS patients in the UK population had a significantly higher risk of presenting with gastric, intestinal, urinary and anorectal disturbances, anxiety, depression, insomnia, fatigue, headache, and various types of pain among patients, up to 10 years prior to the index date [1]. In the Canadian population, fatigue, sleep disorders, anaemia, and pain were more frequent among MS cases relative to controls. The association between MS and anaemia was greater in men, while that between MS and pain increased with age [6]. In our study, we did not observe significant association between MS diagnosis and dermatological or reproductive system problems or head injuries in the total group of patients.

We also analysed the data dividing patients with regard to gender, considering apparent differences in disease epidemiology characteristics between sexes [9,10,11]. We confirmed that female patients diagnosed with MS reported, very often, different complaints and symptoms originating from ophthalmological, musculoskeletal, cardiovascular, digestive, urinary, and laryngological systems compared to the matched healthy female controls from the general population. In addition, headaches and mental disorders also showed a strong association. Interestingly, we found that head injuries were frequently encountered in the female subgroup compared to controls from the general population, while dermatological and reproductive system complaints did not appear significant.

Interestingly, we found clear differences in prodromal symptoms among the male subpopulation. 

For males, the list of overrepresented conditions was shorter and included musculoskeletal, ophthalmic, laryngological, headaches, and cardiovascular system symptoms. Interestingly, reproductive system problems were frequently represented in the male population. There was no significant correlation with MS risk for dermatological, digestive, urinary, and mental complaints. Similarly, head injuries were not significant in that respect as well.

In agreement with previous studies, all of the above signs and symptoms tended to increase in time closer to the full-blown MS presentation [1]. This was significant for headaches, musculoskeletal, laryngological, ophthalmological, urinary tract, and cardiovascular symptoms, as well as for mental disturbances.

Our results shed more light on well-known differences in the epidemiological and clinical characteristics between sexes in multiple sclerosis [11]. One crucial clinical observation is that MS occurs more frequently in women than men, indicating an impact of sex-related factors on susceptibility to MS, which is similar to other autoimmune diseases [9]. These factors include hormonal, genetic, and environmental influences and gene x environment interactions and epigenetic mechanisms. Despite a higher incidence and more robust immune responses, females do not have a poorer prognosis, suggesting a biological resilience mechanism [12]. Indeed, it was repeatedly demonstrated that men are more likely to experience faster disease progression [13,14]. Despite extensive studies, these differences are yet to be fully elucidated. New data in that field bring about genome-wide association studies (GWAS) [15]. One important point highlighted by the genome-wide association studies (GWAS) support the role of microglia in MS development [16], which may contribute to sex bias. In fact, increasing evidence suggests that microglia differ in number and morphology in almost every CNS area and play a central role in sexual differentiation during brain development, influencing processes such as cell proliferation, synaptic connectivity, and cellular physiology [17].

The results of our study extend previous observations. Still, it is impossible to conclude whether they represent risk factors for further development of MS or prove that MS has a long preclinical phase with increased occurrence of prodromal signs and symptoms originating from different systems. In one recent study, it was demonstrated that the levels of serum neurofilament light chain (sNfL) increased 6 years before the clinical MS onset, indicating that MS may have a prodromal phase lasting several years and that neuroaxonal damage occurs already during this phase [18]. Interestingly, another study revealed unique immune signatures of prodromal multiple sclerosis in monozygotic twins [19]. Authors investigated a cohort of identical twin pairs who were discordant for multiple sclerosis. In each twin pair, the immune signatures were remarkably similar, pointing to a strong influence of shared genetic and environmental factors. However, when the authors focused on a subgroup of seemingly healthy cotwins who showed subtle signs of “sub-clinical neuro-inflammation”, they identified a distinct signature of memory T cells. There were no significant differences between males and females included in the study, but there was typical overrepresentation of females in the study group.

## 5. Conclusions

In summary, based on our results, it is likely that the gender-related differences in prodromal signs and symptoms result from different subclinical disease activity and pathological process localization [20]. Further studies are needed to prospectively monitor such differences based on large cohorts of patients with identified risks of MS screened by noninvasive methods.

## 6. Study Limitations

The central nationwide National Health Fund database collected data only from 2009, determining the length of episodes experienced by patients before their first demyelinating events. Such information availability makes it difficult, among other things, to determine the precise moment of an MS disease episode. It was recently analysed in detail and different algorithms have been tested for obtaining epidemiological data for Poland [21]. One possible effect of a limited data scope is that 23.82% of MS cases (patients confirmed to have multiple sclerosis during their first visits in 2016) were identified in patients over 50 years of age, while it is believed that there should be notably fewer of such patients. This fact was neither a consequence of potentially inadequate data quality nor a flawed definition of an MS case. Rather, it was the result of a lack of historical electronic health records and the characteristics of the disease and its treatment at the later stages of progression. We also noticed that older MS patients have less frequent neurologist appointments, are ineligible for disease-modifying-therapy (DMT) programmes, and may only receive rehabilitation and care services, the scale of which in the public health system is limited.

## Figures and Tables

**Table 1:** Sample characteristics at index date.

		All		Female		Male	
		MS-Cases (Group %)	Controls (Group %)	MS-Cases (Group %)	Controls (Group %)	MS-Cases (Group %)	Controls (Group %)
Age	<20	170 (5.56%)	16,728 (5.58%)	121 (5.69%)	11,903 (5.70%)	49 (5.28%)	4825 (5.30%)
	20–29	683 (22.35%)	67,046 (22.35%)	468 (21.99%)	45,942 (21.99%)	215 (23.17%)	21,104 (23.16%)
	30–39	841 (27.52%)	82,538 (27.51%)	585 (27.49%)	57,407 (27.48%)	256 (27.59%)	25,131 (27.58%)
	40–49	634 (20.75%)	62,225 (20.74%)	456 (21.43%)	44,753 (21.42%)	178 (19.18%)	17,472 (19.18%)
	50+	728 (23.82%)	71,463 (23.82%)	498 (23.40%)	48,884 (23.40%)	230 (24.78%)	22,579 (24.78%)
Age	Mean	39.17	39.17	39.13	39.13	39.26	39.26
	SD	14.02	14.03	13.84	13.84	14.45	14.44
Total		3056	300,000	2128	208,889	928	91,111

**Table 2:** Analysed conditions with corresponding ICD-10 codes.

Condition	ICD-10 Codes
Urinary tract diseases	N23.x, N30.x, N39.x
Reproductive system diseases	N34.x, N35.x, N36.x, N40.x, N41.x, N42.x, N43.x, N44.x, N45.x, N46.x, N47.x, N48.x, N49.x, N50.x, N70.x, N72.x, N76.x, N83.x, N86.x, N91.x, N92.x, N93.x, N94.x, N97.x
Ophthalmic diseases	H10.x, H35.x, H40.0, H46.x, H47.x, H52.x, H53.x, H57.x
Musculoskeletal diseases	M12.8, M13.x, M15.x, M17.x, M23.x, M25.x, M41.x, M47.x, M48, M50.x, M51.x, M54.x, M65.x, M70.x
Mental illnesses	F32.x, F33.x, F41.x, F43.x, F48.x
Laryngological diseases	H60.x, H61.x, H65.x, H66.x, H68.x, H81.x, H82.x, H90.x, H91.x, H93.x
Headaches	G43.x, G44.x, R51.x, R52.x
Head injuries	S02.0, S02.1, S02.7, S02.8, S02.9, S04, S06, S07, S09.7, S09.8, S09.9, T02.0, T03.0, T90.5
Dermatological diseases	L02.x, L08.x, L20.x, L21.x, L23.x, L24.x, L25.x, L30.x, L50.x, L60.x, L65.x, L70.x, L98.x
Cardiovascular diseases	I11.x, I25.x, I49.x, I69.x, I70.x
Digestive system diseases	K21.x, K29.x, K30.x, K52.x, K58.x, K59.x, K63.x, K80.x

Codes ended with the ‘.x’ suffix indicate both 3-character codes and their 5-character extensions.

**Table 3:** Examined conditions incidence within subgroups.

	All		Female		Male	
Condition	MS Cases Incidence	Controls Incidence	MS Cases Incidence	Controls Incidence	MS Cases Incidence	Controls Incidence
Urinary tract diseases	0.230	0.192	0.284	0.237	0.108	0.090
Reproductive system diseases	0.392	0.380	0.499	0.495	0.147	0.118
Ophthalmic diseases	0.499	0.351	0.541	0.386	0.402	0.270
Musculoskeletal diseases	0.592	0.482	0.624	0.498	0.517	0.447
Mental illnesses	0.218	0.180	0.264	0.210	0.112	0.109
Laryngological diseases	0.249	0.189	0.269	0.199	0.203	0.168
Headaches	0.307	0.168	0.356	0.197	0.195	0.101
Head injuries	0.016	0.013	0.014	0.009	0.022	0.022
Dermatological diseases	0.406	0.391	0.442	0.426	0.324	0.312
Cardiovascular diseases	0.196	0.142	0.199	0.141	0.188	0.143
Digestive system diseases	0.364	0.340	0.396	0.358	0.289	0.297

## Data Availability

Restrictions apply to the availability of this dataset. Dataset contains administrative health data which was obtained with the permission of Ministry of Health of the Republic of Poland from the National Health Fund (NFZ) electronic databases. Aggregated data on multiple sclerosis from the NFZ registers could be accessed through interactive reports published by the Department of Analysis and Strategy of Polish Ministry of Health at https://basiw.mz.gov.pl/index.html#/visualization?id=3454 (accessed on 23 August 2021). and https://basiw.mz.gov.pl/index.html#/visualization?id=3700 (accessed on 23 August 2021).

## Supplementary Materials

The following are available online at https://www.mdpi.com/article/10.3390/jcm10173821/s1, Figure S1: Urinary tract diseases temporal Risk Ratio estimates, Figure S2: Reproductive system diseases temporal Risk Ratio estimates, Figure S3: Ophthalmic diseases temporal Risk Ratio estimates, Figure S4: Musculoskeletal diseases temporal Risk Ratio estimates, Figure S5: Mental illnesses temporal Risk Ratio estimates, Figure S6: Laryngological diseases temporal Risk Ratio estimates, Figure S7: Headaches temporal Risk Ratio estimates, Figure S8: Head injuries temporal Risk Ratio estimates, Figure S9: Dermatological diseases temporal Risk Ratio estimates, Figure S10: Cardiovascular diseases temporal Risk Ratio estimates, Figure S11: Digestive system diseases temporal Risk Ratio estimates, Table S1: Risk Ratio estimates within subgroups. Table S1, with detailed information on risk ratio estimates, as presented in Figure 1, is disclosed in the article. Figures S1–S11 depicting all temporal risk ratio estimates are briefly discussed in the Section 3.
