# Supplementary material for: Gender-Related Differences in Prodromal Multiple Sclerosis Characteristics: A 7-Year Observation Study
Source: J Clin Med. 2021 Aug 26;10(17):3821. doi: 10.3390/jcm10173821 (PMC8432063; doi:10.3390/jcm10173821)
Supplement: Supplementary file 1 [file jcm-10-03821-s001.zip › figureS1-11.pdf]

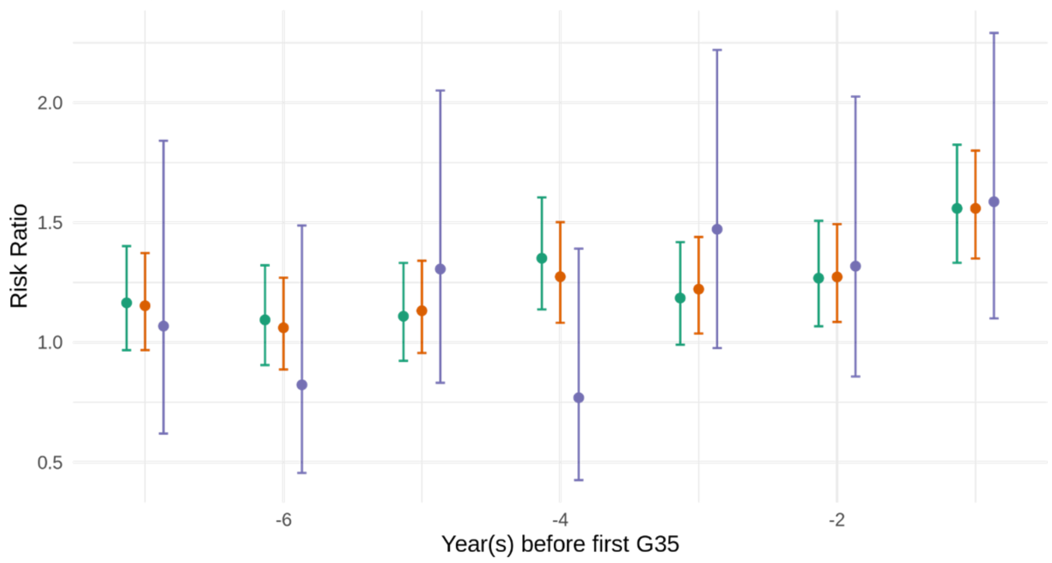

Figure S1 Urinary Tract Diseases

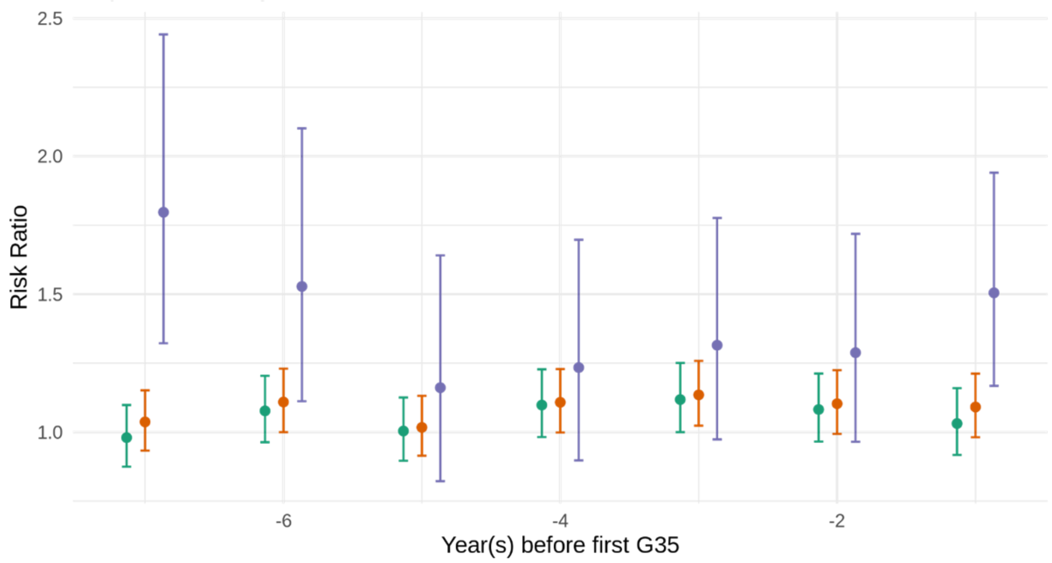

Figure S2 Reproductive_System_Diseases

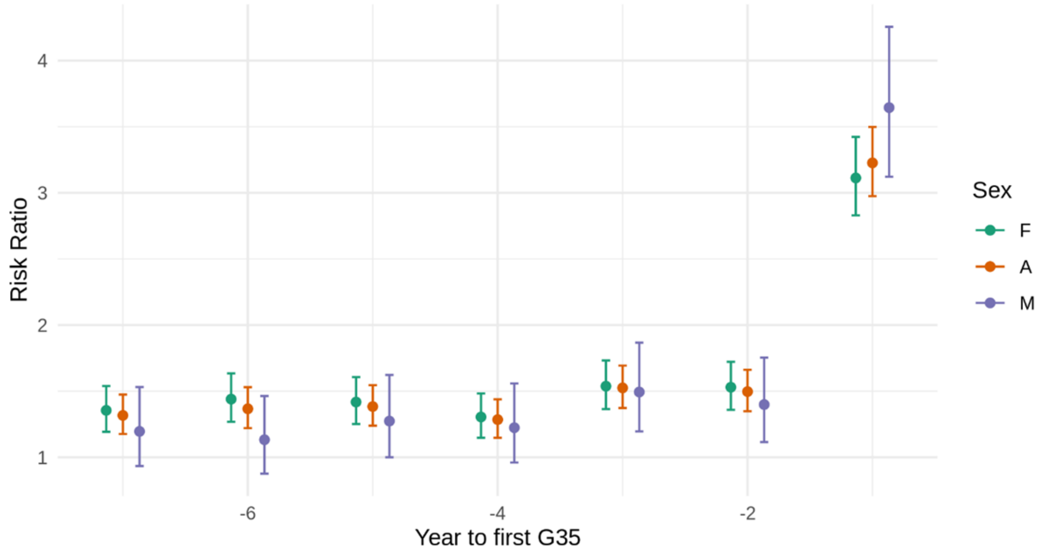

Figure S3 Ophtalmic Diseases

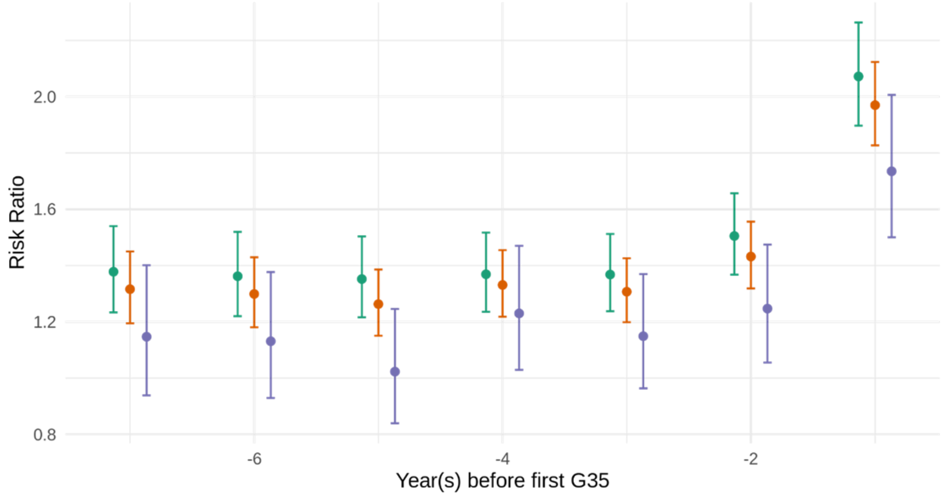

Figure S4 Musculoskeletal Diseases

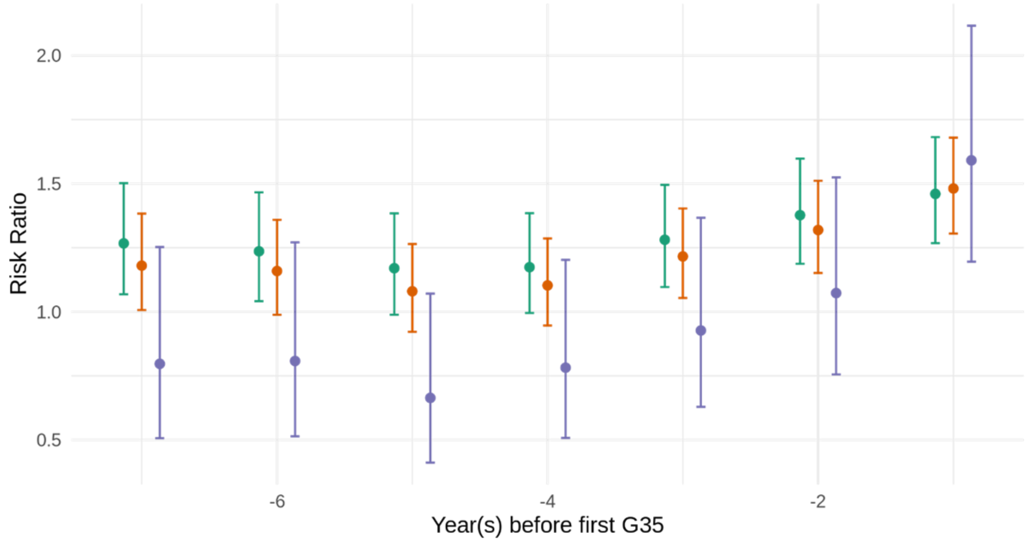

Figure S5 Mental_illnesses

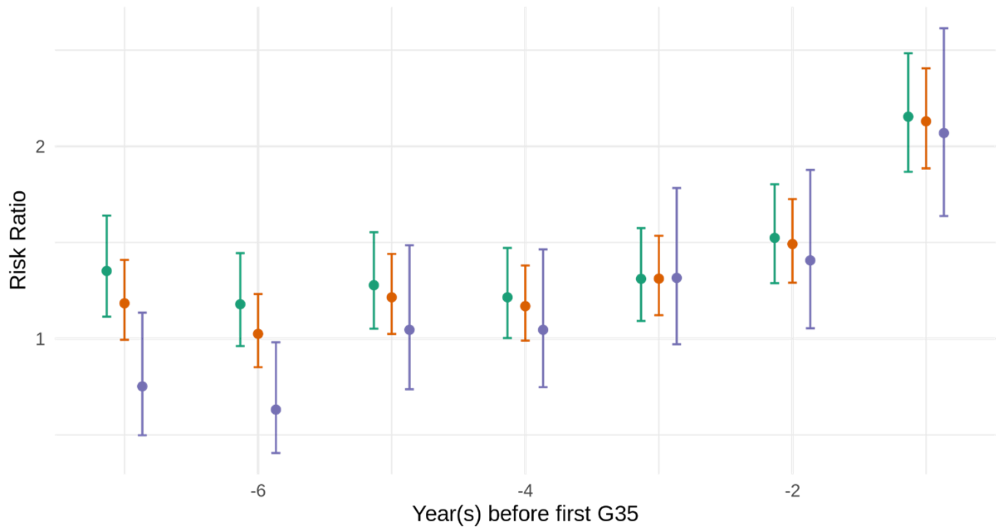

Figure S6 Laryngological Diseases

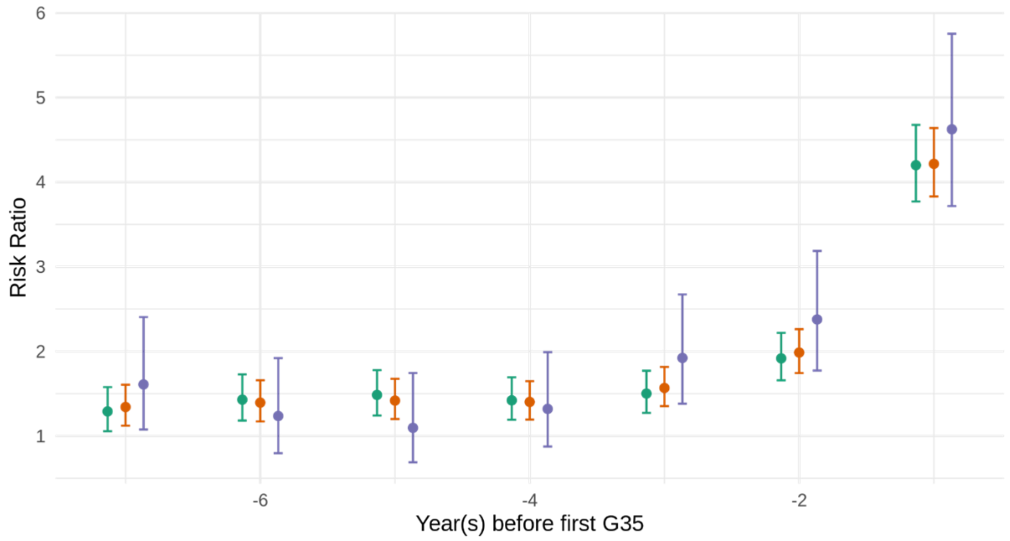

Figure S7 Headaches

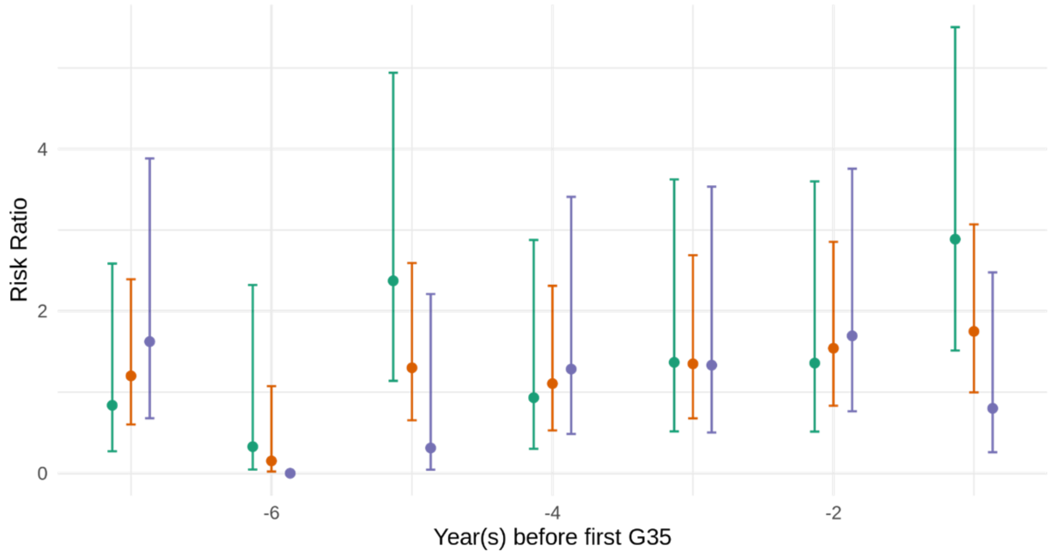

Figure S8 Head Injuries

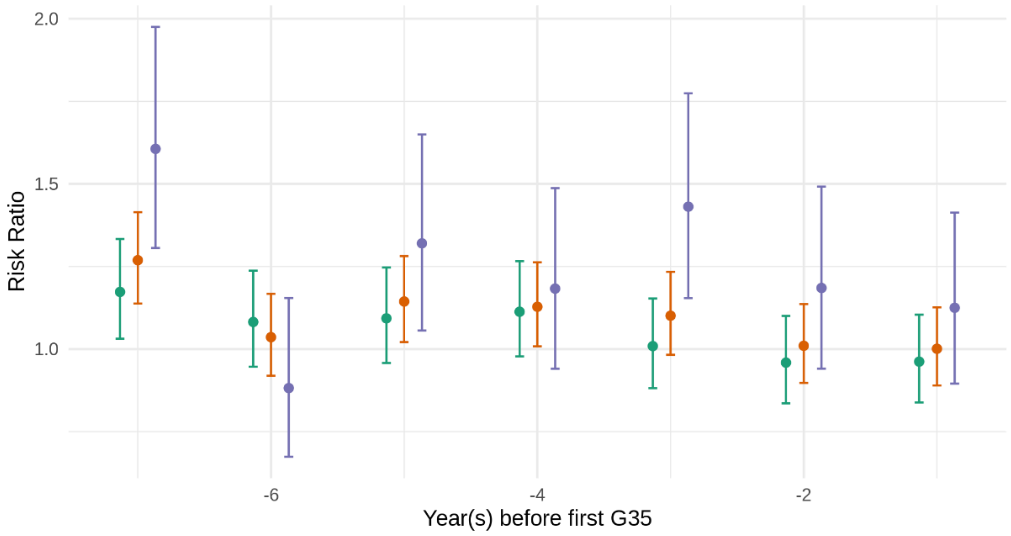

Figure S9 Dermatological Diseases

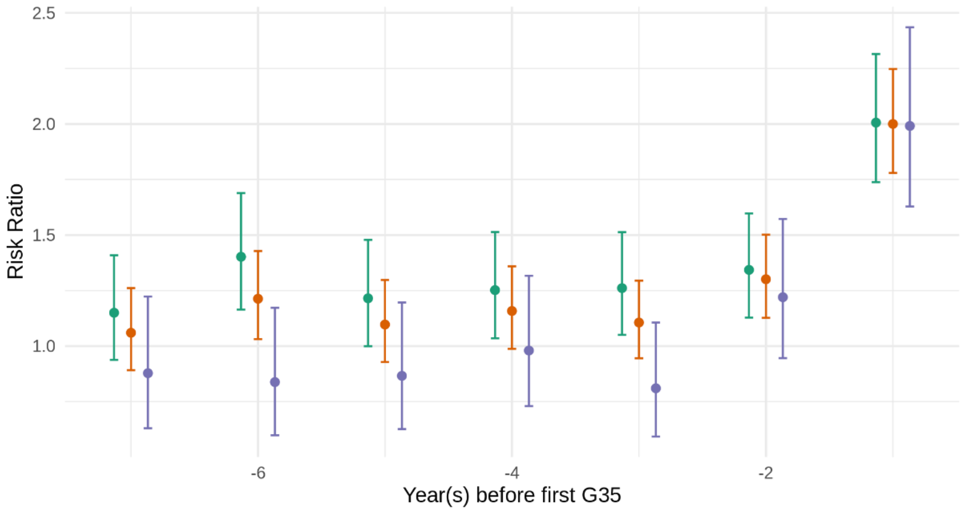

Figure S10 Cardiovascular Diseases

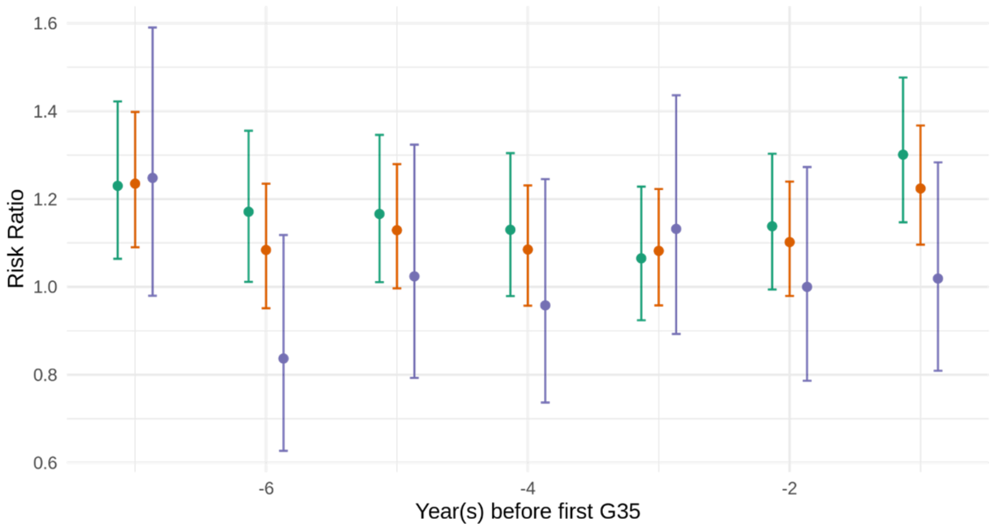

Figure S11 Digestive System Diseases
