# Supplementary material for: Gender-Related Differences in Prodromal Multiple Sclerosis Characteristics: A 7-Year Observation Study
Source: J Clin Med. 2021 Aug 26;10(17):3821. doi: 10.3390/jcm10173821 (PMC8432063; doi:10.3390/jcm10173821)
Supplement: Supplementary file 1 [file jcm-10-03821-s001.zip › Table_S1.pdf]

Table S1. Risk Ratio estimates within subgroups

Condition	All			Female			Male		
	Risk Ratio (RR)	95% RR CI	*p*-value	Risk Ratio (RR)	95% RR CI	*p*-value	Risk Ratio (RR)	95% RR CI	*p*-value
Urinary tract diseases	1.255	(1.155, 1.365)	< 0.001	1.274	(1.16, 1.399)	< 0.001	1.223	(0.995, 1.503)	0.057
Reproductive system diseases	1.049	(0.976, 1.128)	0.196	1.016	(0.933, 1.105)	0.727	1.284	(1.071, 1.538)	0.008
Ophthalmic diseases	1.830	(1.706, 1.964)	< 0.001	1.862	(1.711, 2.027)	< 0.001	1.808	(1.587, 2.060)	< 0.001
Musculoskeletal diseases	1.548	(1.440, 1.663)	< 0.001	1.666	(1.527, 1.818)	< 0.001	1.322	(1.163, 1.502)	< 0.001
Mental illnesses	1.266	(1.163, 1.379)	< 0.001	1.339	(1.216, 1.473)	< 0.001	1.030	(0.841, 1.262)	0.751
Laryngological diseases	1.411	(1.301, 1.531)	< 0.001	1.477	(1.343, 1.625)	< 0.001	1.251	(1.067, 1.467)	0.007
Headaches	2.176	(2.016, 2.348)	< 0.001	2.231	(2.043, 2.436)	< 0.001	2.132	(1.815, 2.505)	< 0.001
Head injuries	1.252	(0.948, 1.653)	0.128	1.508	(1.055, 2.157)	0.031	0.996	(0.641, 1.547)	1.000
Dermatological diseases	1.065	(0.991, 1.144)	0.090	1.067	(0.980, 1.162)	0.134	1.060	(0.925, 1.215)	0.413
Cardiovascular diseases	1.469	(1.344, 1.605)	< 0.001	1.508	(1.356, 1.676)	< 0.001	1.382	(1.173, 1.628)	< 0.001
Digestive system diseases	1.109	(1.031, 1.194)	0.006	1.172	(1.075, 1.278)	< 0.001	0.963	(0.836, 1.109)	0.613

RR – Risk Ratio; p-value – Fisher's exact test p-value; 95% RR CI – 95% Risk Ratio Confidence Interval
